# In vitro comparison of the imaging properties of dental materials in soft tissue using ultrasonography and cone beam computed tomography

**DOI:** 10.1038/s41598-025-29235-4

**Published:** 2025-12-29

**Authors:** Çiğdem Şeker, Ebru Yüksel Kaya, Gediz Geduk, Orhan Cicek, Murat İçen

**Affiliations:** 1 Faculty of Dentistry, Dentomaxillofacial Radiology Department , Zonguldak Bülent Ecevit University , Zonguldak, Turkey; 2 Iskenderun Oral and Dental Health Center , Hatay, Turkey; 3 Faculty of Dentistry, Department of Orthodontics , Zonguldak Bülent Ecevit University , Zonguldak, Turkey; 4 Faculty of Dentistry, Department of Dentomaxillofacial Radiology, Nevşehir Hacı Bektaş Veli University, Nevşehir, Turkey

**Keywords:** Dental materials, Soft tissue foreign bodies, Cone beam computed tomography, Ultrasonography, Imaging properties, Diseases, Health care, Materials science, Medical research

## Abstract

Accurate imaging of displaced dental materials (DMs) in soft tissues is critical for diagnosis, treatment planning, and preventing complications. The aim of this study was to compare the cone beam computed tomography (CBCT) and ultrasonography (USG) in detecting DMs embedded in soft tissues, as well as to characterize their imaging features. Fifteen DMs used in restorative, endodontic, orthodontic, and prosthetic procedures were placed in the alveolar mucosa and muscles. CBCT and USG images of DMs were obtained. Two dentomaxillofacial radiologists independently evaluated the images for detectability, radiopacity, echogenicity, and artifact presence. USG demonstrated superior diagnostic accuracy, sensitivity, and specificity compared to CBCT, particularly for low-radiopacity and superficial materials, such as paper points, wax, alginate, and acrylic. While CBCT effectively visualized high-density materials, such as amalgam, metal seramic crowns, Ni-Ti wires, and metal brackets, it failed to detect low-density objects, like paper points and wax. Intra- and interobserver agreements were excellent for both modalities. Posterior acoustic shadowing and comet-tail patterns were observed in USG. It was concluded that USG is effective for detecting superficial, low-radiopacity DMs, while CBCT excels in evaluating high-density materials, highlighting their complementary roles in managing foreign bodies in hard and soft oral tissues. These results may help clinicians select appropriate imaging modalities based on material characteristics, thereby improving diagnostic accuracy and reducing unnecessary radiation exposure. In addition, the study provides a useful reference for clinicians by outlining the ultrasonographic features of DMs.

**Supplementary Information:**

The online version contains supplementary material available at 10.1038/s41598-025-29235-4.

## Introduction

The anatomical complexity of the head and neck region, with its numerous vital structures, necessitates meticulous care during dental procedures. In iatrogenic and traumatic situations, such as accidents, dental materials (DMs) can displace into soft tissues, posing a substantial risk of serious complications. An accurate diagnosis and timely intervention are essential to effectively treat and prevent such adverse outcomes^1–3^.

Delayed intervention may result in complications such as infection, impaired wound healing, persistent inflammation, and functional impairment. The soft tissues of the head and neck, comprising muscles, blood vessels, nerves, and connective tissues, should be preserved to ensure patient safety and prevent long-term functional impairments^3–6^. The displacement of materials used in dental treatments into the soft tissues of the head and neck region may lead to negative consequences for the reasons described^1–7^.

The evaluation of soft tissue foreign bodies can involve various imaging techniques, including panoramic radiography, magnetic resonance imaging (MRI), ultrasonography (USG), and cone beam computed tomography (CBCT), in addition to thorough clinical examination and detailed patient history. CBCT, an imaging method that is becoming increasingly common in dentistry, provides high-resolution and detailed images to help determine the exact placement of the material, whereas panoramic radiographs may be inadequate due to superimposition. However, the radiation exposure associated with CBCT should also be taken into consideration. Additionally, the soft tissue image quality is poor due to the low contrast resolution of CBCT. Medical CT devices provide higher quality soft tissue images due to their high contrast resolution^6–15^.

On the other hand, USG has become an integral component of diagnostic imaging due to its ease of use, absence of ionizing radiation, and high reproducibility. While numerous studies have investigated the application of USG for detecting soft tissue foreign bodies in ex vivo and in vitro models, there is a notable paucity of research specifically evaluating its diagnostic accuracy for DMs. Most of the existing studies have focused on non-dental foreign bodies, such as stone, wood, or glass fragments, primarily assessing their imaging characteristics rather than clinical applicability^1,3,4,6–15^. Consequently, the evidence supporting the use of USG for precise localization and characterization of DMs within soft tissues remains limited, highlighting the need for systematic investigation in this area^1,3,7,12^.

This gap makes it challenging for clinicians to have a clear roadmap regarding which imaging modality would be most suitable in specific clinical scenarios. In this context, directly comparing the performance of two widely used methods, CBCT and USG, in detecting DMs could provide evidence-based data to support clinical decision-making, enhance diagnostic efficiency, and minimize unnecessary radiation exposure.

The aim of this study was to characterize the imaging features (visibility, echogenicity, artifact type) of various DMs embedded in soft tissue using CBCT and USG under in vitro conditions, and to provide pilot data on the comparative imaging trends (sensitivity and specificity values) of these modalities. It is important because it has the potential to provide clinicians with evidence-based guidance for optimal diagnosis and management by directly comparing two widely accessible imaging techniques.

## Results

**Fig. 1:**
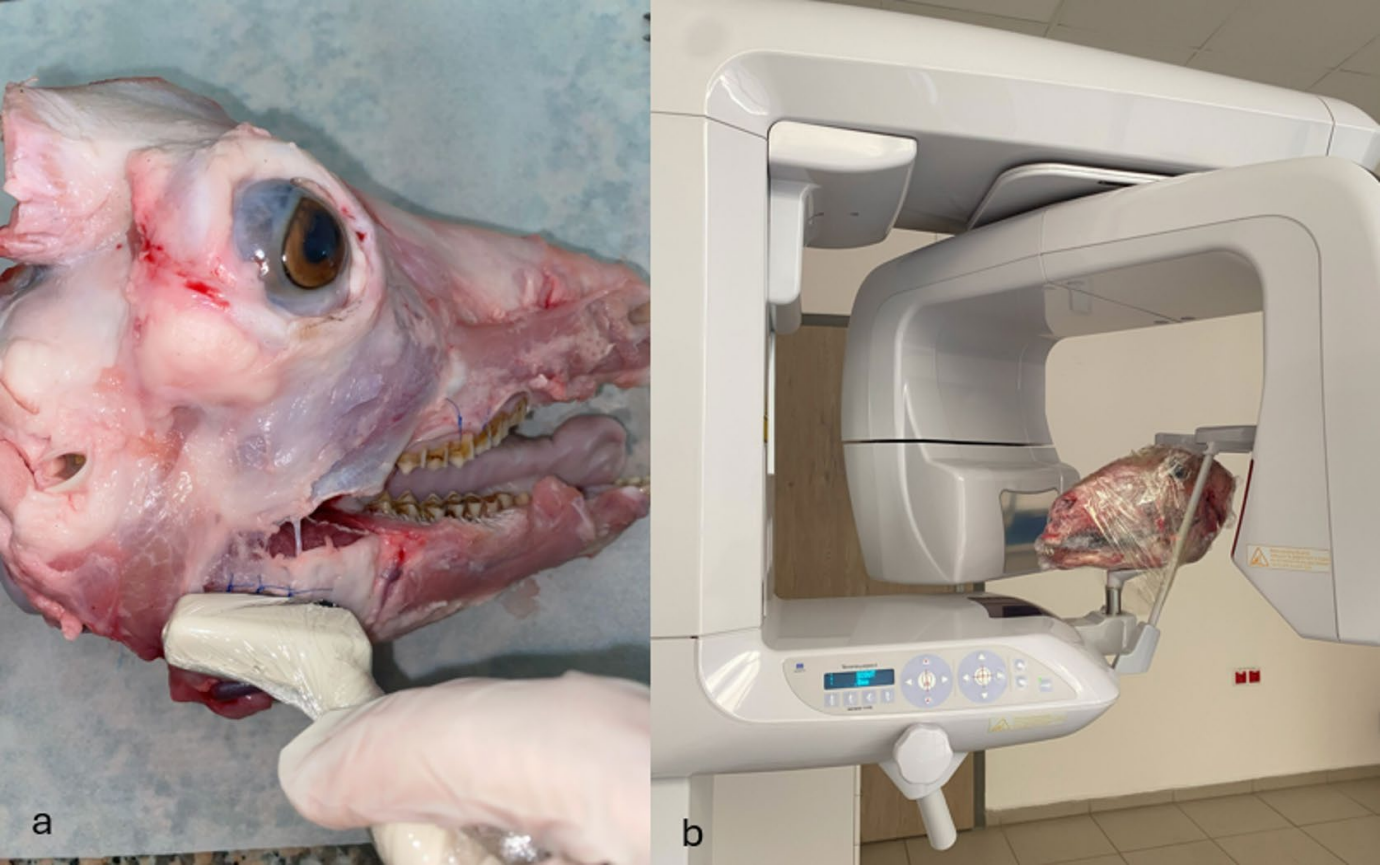
Sheep head used to obtain images; (**a**) USG imaging and (**b**) CBCT imaging.

**Fig. 2:**
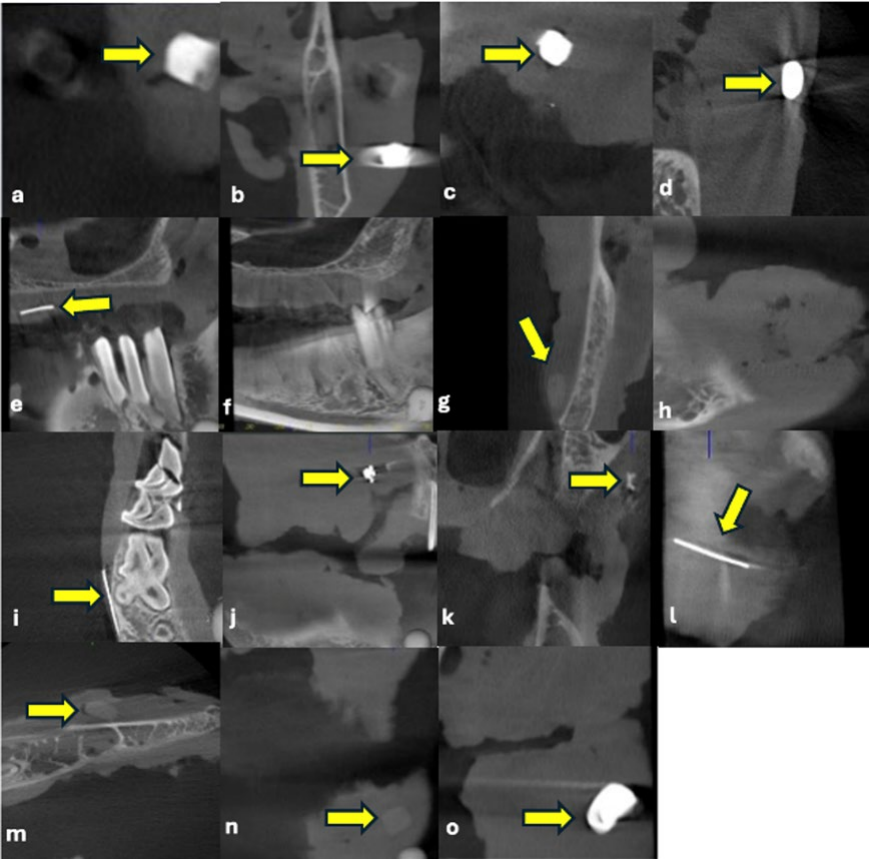
CBCT imaging of DMs; (**a**) Composite, (**b**) Amalgam, (**c**) Glass-ionomer cement, (**d**) Zincopolycarboxylate cement, (**e**) Gutta percha, (**f**) Paper point (not visible), (**g**) Acrylic, (**h**) Wax (not visible), (**i**) Needle, (**j**) Stainless steel bracket, (**k**) Ceramic bracket, (**l**) Ni-Ti wires, (**m**) Alginate, (**n**) Acrylic tooth, and (**o**) Metal ceramic crown.

**Fig. 3:**
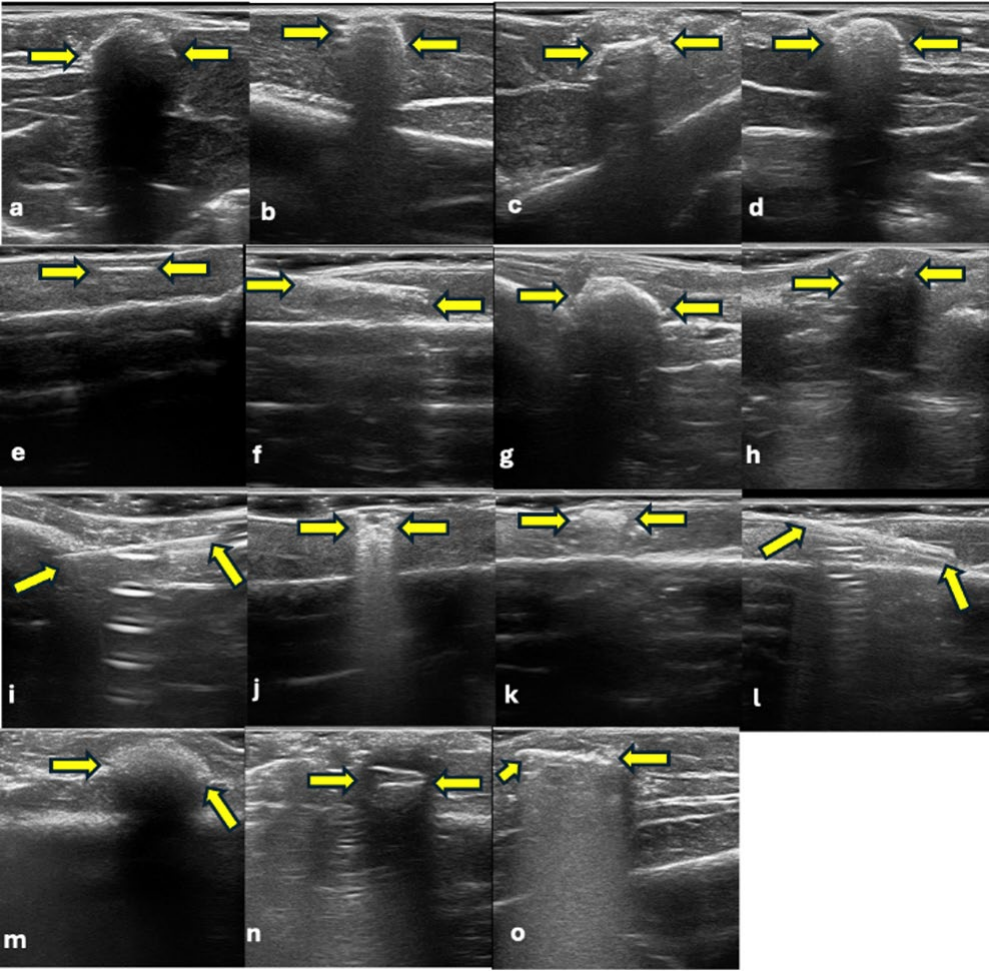
Ultrasonographic visualization of DMs; (**a**) Composite, (**b**) Amalgam, (**c**) Glass-ionomer cement, (**d**) Zinc polycarboxylate cement, (**e**) Gutta percha, (**f**) Paper point, (**g**) Cold-curing acrylic, (**h**) Dental pink wax, (**i**) Injector needle, (**j**) Stainless steel bracket, (**k**) Ceramic bracket, (**l**) Ni-Ti wires, (**m**) Alginate, (**n**) Acrylic tooth, and (**o**) Metal ceramic crown.

Table 1 shows the TP, FP, TN, FN values ​​for each modality. As shown in Table 2, both the intra- and interobserver agreements for the CBCT and USG evaluations were excellent (κ = 0.919–1.000, *p* < 0.001). Almost perfect agreement was achieved for both visibility/echogenicity and artifact presence in USG and CBCT. These findings demonstrate the reliability and reproducibility of the assessment method used in this study. As shown in Table 3, notably, USG outperformed CBCT, particularly in detecting non-opaque materials. These findings support the use of USG as an advanced imaging modality in clinical practice, serving as a complement to CBCT when necessary (Figs. 1, 2 and 3). These pilot findings provided useful information for sample size estimation and protocol refinement for the subsequent study.

**Table 1:** 2 × 2 contingency tables for CBCT and USG Assessment.

CBCT (all observers and reads):	Gold standard positive (*n* = 15)	Gold standard negative (*n* = 1)
Test positive	TP = 13	FP = 0
Test negative	FN = 2	TN = 1
USG (all observers and reads):	Gold standard positive (*n* = 15)	Gold standard negative (*n* = 1)
Test positive	TP = 15	FP = 0
Test negative	FN = 0	TN = 1

**Table 2:** κ values for intra and interobserver agreement and p values.

	Intraobserver Cohen’s κ values		Interobserver Cohen’s κ values		For interobserver
	1 st observer 1 st and 2nd reading	2nd observer 1 st and 2nd reading	1 st reading	2nd reading	Observed agreement (%)
USG (visibility and echogenicity)	1	1	1	1	100% (16/16 cases)
p	< 0.001^*^	< 0.001^*^	< 0.001^*^	< 0.001^*^	
USG (artifact)	1	1	0.919	0.919	
p	< 0.001^*^	< 0.001^*^	< 0.001^*^	< 0.001^*^	
CBCT (visibility and density)	1	1	1	1	93.8% (15/16 cases)
p	< 0.001^*^	< 0.001^*^	< 0.001^*^	< 0.001^*^	

*p* significance value,^*^p < 0.05

**Table 3:** Preliminary diagnostic accuracy findings reflecting the methods’ material-specific tendencies.

		Sensitivity (95% CI)	Specificity (95% CI)	PPV (95% CI)	NPV (95% CI)	Diagnostic accuracy (95% CI)
USG	1 st observer-1st reading	100% (78.2–100)	100% (2.5–100)	100% 78.2–100)	100% (2.5–100)	100% (79.4–100)
	1 st observer-2nd reading	100% (78.2–100)	100% (2.5–100)	100% (78.2–100)	100% (2.5–100)	100% (79.4–100)
	2nd observer-1st reading	100% (78.2–100)	100% (2.5–100)	100% (78.2–100)	100% (2.5–100)	100% (79.4–100)
	2nd observer-2nd reading	100% (78.2–100)	100% (2.5–100)	100% (78.2–100)	100% (2.5–100)	100% (79.4–100)
CBCT	1 st observer-1st reading	86.7% (59.5–98.3)	100% (2.5–100)	100% (75.3–100)	33.3% (0.8–90.6)	87.5% (61.7–98.4)
	1 st observer-2nd reading	86.7% (59.5–98.3)	100% (2.5–100)	100% (75.3–100)	33.3% (0.8–90.6)	87.5% (61.7–98.4)
	2nd observer-1st reading	86.7% (59.5–98.3)	100% (2.5–100)	100% (75.3–100)	33.3% (0.8–90.6)	87.5% (61.7–98.4)
	2nd observer-2nd reading	86.7% (59.5–98.3)	100% (2.5–100)	100% (75.3–100)	33.3% (0.8–90.6)	87.5% (61.7–98.4)

*PPV* positive predictive value, *NPV* negative predictive value, *CI* Confidence Interval.

Table 4 presents detailed information on the CBCT visibility and radiopacity, as well as USG visibility, echogenicity, and artifact presence for each DM. In CBCT, paper point and wax were not visible, whereas all materials were detected with USG. In CBCT, acrylic, ceramic bracket, alginate, and acrylic tooth were classified as “slightly opaque.” In USG, most materials appeared hyperechoic, with alginate being the only material identified as isoechoic. Artifact patterns varied depending on the material, with posterior acoustic shadowing and comet-tail artifacts being the most frequently observed. This table clearly shows the differences in performance and imaging characteristics between the two modalities based on material type (Figs. 2 and 3).

**Table 4:** Ultrasonographic and radiographic features of DM.

Material	CBCT		USG		
	Visibility	Density	Visibility	Echogenicity	Artifact
Composite	Visible	Opaque	Visible	Hyperechoic	Posterior acoustic shadowing
Amalgam	Visible	Opaque	Visible	Hyperechoic	Comet tail and posterior acoustic shadowing
GIC	Visible	Opaque	Visible	Hyperechoic	Comet tail
Zinc polycarboxylate cement	Visible	Opaque	Visible	Hyperechoic	Comet tail and posterior acoustic shadowing
Gutta percha	Visible	Opaque	Visible	Hyperechoic	Not visible
Paper point	Not visible	Not visible	Visible	Hyperechoic	Not visible
Acrylic	Visible	Slightly opaque	Visible	Hyperechoic	Posterior acoustic shadowing
Wax	Not visible	Not visible	Visible	Hyperechoic	Posterior acoustic shadowing
Needle	Visible	Opaque	Visible	Hyperechoic	Comet tail
Stainless steel bracket	Visible	Opaque	Visible	Hyperechoic	Comet tail
Ceramic bracket	Visible	Slightly opaque	Visible	Hyperechoic	Not visible
Ni-Ti wires	Visible	Opaque	Visible	Hyperechoic	Comet tail
Alginate	Visible	Slightly opaque	Visible	Isoechoic	Posterior acoustic shadowing
Acrylic tooth	Visible	Slightly opaque	Visible	Hyperechoic	Posterior acoustic shadowing
Metal ceramic crowns	Visible	Opaque	Visible	Hyperechoic	Comet tail

## Discussion

Accurate imaging is essential in dentomaxillofacial radiology, particularly for the detection of DMs embedded in soft tissues. This study compared CBCT and USG in their ability to visualize a range of commonly used DMs under in vitro conditions, providing insight into the relative strengths and limitations of each modality.

The detection performance of imaging modalities depends on the physical principles underlying the technology used. In CBCT, visibility is determined by a material’s ability to attenuate X-rays, known as radiopacity, and the difference in density between the material and the surrounding tissue. Due to their high X-ray attenuation, materials containing metal (e.g., amalgam, metal-ceramic crowns, and Ni-Ti wires) are clearly visualized. In contrast, low-density organic materials, such as paper point and wax, were not detectable on CBCT due to their low X-ray absorption. Visibility in USG depends on the difference in acoustic impedance between the material and the surrounding tissue. A high impedance mismatch results in a strong reflection of ultrasound waves, producing a hyperechoic appearance^6,9,10,16^. In our study, most materials appeared hyperechoic, whereas alginate, which has a density close to water, was evaluated as isoechoic. This can be attributed to the acoustic properties of alginate being similar to those of the surrounding soft tissue.

Artifacts can significantly impact the diagnostic accuracy of imaging methods. In CBCT, the most common artifacts are beam hardening, streaking, and scattering. High-density metal-containing materials (e.g., amalgam, metal-ceramic crowns, and Ni-Ti wires) strongly attenuate X-rays. This causes beam hardening, resulting in gray tone loss, reduced contrast, and masking of surrounding details. Streak artifacts are often seen around thin metallic structures and may obscure low-density or small foreign bodies within the shadow of metal artifacts. This can lead to false negatives. In USG, artifacts such as posterior acoustic shadowing and comet-tail patterns can assist in detecting foreign bodies, but they can also mislead interpretation. Shadowing is pronounced in materials with high acoustic impedance differences, while the comet-tail artifacts are typically observed around metallic materials. However, these findings may be faint or absent in small or low-reflectivity objects, and normal structures, such as calcifications or fibrotic tissue, can mimic them. Furthermore, prior surgical intervention may cause air to accumulate within the tissue, which complicates the imaging process and can result in erroneous findings. Accurate interpretation requires considering the physical properties of the material, the surrounding tissue, and the device settings. MAR algorithms in CBCT and multiplanar probe positioning in USG can improve diagnostic accuracy^5,9,12,14,16,17^.

One notable finding of this study is that while all of the examined DMs were detectable using USG, CBCT failed to identify paper points and wax. This finding is consistent with existing literature highlighting the limitations of CBCT in visualizing non-opaque materials. Demiralp et al. reported that low-density materials were more effectively visualized using USG^5^. Aras et al. found that USG detected superficial foreign bodies with low radiopacity in body tissues more effectively than CT and conventional plain radiography^6^. Valizadeh et al. recommended using USG to diagnose foreign bodies in superficial soft tissues without surrounding bone. The main difference between the studies is the type of foreign bodies examined. While previous studies frequently examined various objects, such as glass, wood chips, and asphalt, our study focused solely on DMs.

Kaygısız Yiğit et al. evaluated 26 foreign bodies and DMs in bovine gelatin using panoramic radiography and USG. Materials such as paper points and wax, which were undetectable on CBCT in our study, were also not visible on panoramic radiographs. The echogenicity of wax, alginate, and glass ionomer cement differed in USG from our observations^1^. A comparable study was conducted by Çağlayan et al.^7^, in which acrylic, alginate, wax, and paper points were not detectable on panoramic radiography. In contrast, in our study, alginate and acrylic appeared slightly radiopaque. Only alginate showed a difference in the USG examination. These studies evaluating the acoustic behavior of DMs frequently observed posterior acoustic shadowing, which is consistent with our findings^1,7^. These variations in results are likely due to the different brands and origins of the materials used. Furthermore, while our study employed a sheep head to simulate a more natural scenario, the materials in the other studies were embedded in gelatin. Since bovine gelatin provides a more homogeneous environment with a higher water content, it may transmit and reflect ultrasound waves differently^18^. This could explain the discrepancies observed in the echogenicity of the material.

However, the superior performance of CBCT in our study is not universal and should be interpreted in the context of material properties. While studies by Isman O and Isman E (evaluating orthodontic materials)^9^ and Shokri et al. (using wood, glass, and stone)^8^ recommended CBCT as the most effective modality, they primarily assessed highly radiopaque objects. This highlights a key distinction: CBCT excels with radiopaque materials like metal and glass due to strong X-ray attenuation, but its performance is inherently limited for low-density objects. Conversely, Shokri et al. reported a relatively low sensitivity of 33.33% for USG, a finding that can be attributed to fundamental physical principles^8^. USG detection relies on a sufficient difference in acoustic impedance between the foreign body and the surrounding tissue; if this impedance contrast is low, detectability drops. Furthermore, material size, depth, and tissue heterogeneity significantly influence USG performance, with signal attenuation and artifacts from bone or air interfaces posing challenges. An important caveat from Javadrashid et al. corroborates this nuanced view, noting that wood particles larger than 0.5 mm could only be visualized by USG^13^, underscoring that USG retains a unique diagnostic niche even for materials often considered radiolucent. Finally, it is crucial to note that these comparative performances are often observed under controlled in vitro conditions, where factors like tissue homogeneity may enhance detectability compared to the clinical setting^8,9,13^.

The type of imaging methods used to detect foreign bodies should be based on the material type, anatomical location, and patient-specific factors. Although conventional X-rays are fast and cost-effective, they may be insufficient for visualizing certain materials. CT is considered the gold standard due to its wide applicability and high accuracy; however, its radiation exposure remains a significant disadvantage. USG is effective for evaluating superficial tissues and useful in intraoperative settings, but its accuracy depends on the operator. While MRI offers superior soft tissue contrast, it has limitations regarding accessibility and cost. In addition, the presence of metallic foreign bodies should be assessed using X-ray or CT prior to MRI because ferromagnetic objects pose a security risk. Therefore, the most appropriate imaging modality should be selected based on the specific clinical requirements of each case^15^.

Several methodological limitations warrant consideration when applying these results. Most notably, the postmortem setting cannot replicate the dynamic physiological environment of living tissues, where factors such as blood flow and inflammation could alter imaging characteristics. Furthermore, while fifteen different DMs were evaluated, the use of only a single positive and negative sample for each may have led to an overestimation of sensitivity and specificity. Although confidence intervals were calculated, the limited number of negative cases resulted in extremely wide intervals, limiting their practical utility. Consequently, the diagnostic accuracy metrics should be interpreted as pilot findings to guide future confirmatory studies with larger, more balanced sample sizes. Additional limitations pertain to the assessment methodology. The evaluations of radiopacity and echogenicity were based on qualitative methods, which, despite being widely used in the literature, are inherently subjective and introduce potential for observer variability. Future research would benefit from integrating objective, quantitative techniques, such as digital image processing of pixel intensities, to enhance the accuracy and reproducibility of such assessments. Finally, the very high inter- and intra-observer agreement (as indicated by the Kappa values) may be partly attributable to a prevalence effect, where an unbalanced distribution of findings in the sample can inflate agreement estimates, particularly for rare conditions.

A key strength of this study—the inclusion of 15 commonly used dental materials in a realistic postmortem model—directly informs its clinical implications. The direct comparison of CBCT and USG elucidates their complementary roles, offering clear guidance for practitioners. USG emerges as a reliable, first-line tool for detecting non-opaque and superficial DMs, providing a means to reduce unnecessary radiation exposure. Conversely, CBCT remains indispensable for evaluating high-density DMs and clarifying the precise spatial relationships of deeply embedded foreign bodies due to its high spatial resolution and 3D capabilities. Consequently, the choice of imaging modality should be guided by clinical suspicion: USG is often preferred for initial evaluation of soft-tissue foreign bodies, leveraging its portability, cost-effectiveness, and safety. CBCT, however, should be reserved for cases requiring detailed assessment of radiopaque objects or complex spatial anatomy, where its clear diagnostic benefit justifies the associated radiation exposure.

## Methods

### Preparation of samples

For this study, one sheep’s head purchased from a butcher was used. Procedures were performed on the sample one day after death. All images were taken on the same day. The selected DMs were used without standardizing their dimensions in order to simulate natural conditions^7,9^. To increase transparency and assist reproducibility, the actual dimensions of each sample have been recorded and are provided in Table 5.

**Table 5:** Dimensions of the DMs used in the study.

Material	Size (mm)
1. Composite	5 × 5
2. Amalgam	5 × 5
3. GIC	5 × 5
4. Zinc polycarboxylate cement	5 × 5
5. Gutta-percha #40	10
6. Paper point #35	10
7. Cold-curing acrylic	5 × 5
8. Wax	5 × 5
9. Syringe with hypodermic needle 0.40 mm x 50 mm 27 G	16
10. Stainless steel bracket #24	4 × 3
11. Ceramic bracket #24	4 × 3
12. Ni-Ti wires	16
13. Alginate	7 × 5
14. Acrylic tooth	7 × 5
15. Metal ceramic crowns	9.5 × 7

A pocket was prepared with a scalpel, and the materials were placed randomly in different areas. The sockets of the materials were sutured to prevent displacement. In the inferior part of the left masseter muscle, horizontal incisions were made on the muscle surface, and the muscle fibers were released by blunt dissection in the vertical direction. Three intramuscular tunnels were then created at the level of the mandibular angle, and amalgam, metal-ceramic crown, and acrylic teeth were placed in these tunnels from anterior to posterior, respectively. In the superior part of the left masseter muscle, three intramuscular tunnels were created at the level of the posterior maxilla, and glass ionomer cement, zinc polycarboxylate cement, and composite resin were placed from anterior to posterior, respectively. In the inferior part of the right masseter muscle, horizontal incisions were made on the muscle surface at the level of the mandibular angle, and the muscle fibers were released by blunt dissection in the vertical direction. Three intramuscular tunnels were prepared from anterior to posterior, and wax, acrylic, and alginate were placed in these tunnels from anterior to posterior, respectively. Similarly, three intramuscular tunnels were created in the superior part of the right masseter muscle at the level of the posterior maxilla using the same technique, and Ni-Ti archwire, ceramic bracket, and stainless steel bracket were placed in these tunnels, respectively. In addition, two full-thickness mucoperiosteal flaps were elevated bilaterally on the lingual surfaces of the right and left molars via horizontal incisions approximately 2 mm apical to the gingival sulcus; a paper point was placed on the right side, and gutta-percha was placed on the left side. On the buccal surfaces of the right molars, a full-thickness mucoperiosteal flap was elevated via a horizontal incision approximately 2 mm apical to the gingival sulcus, and a needle was inserted. The tunnels and flaps were prepared to a depth sufficient to allow the materials to be placed without creating tension on the tissue; however, millimetric depth measurements were not taken. All materials were placed and sutured during the same surgical session. The posterior region of the right masseter muscle was used as a negative control, and no material was placed in this region.

This setup clearly differentiated the presence or absence of DMs, enabling reliable comparisons and validations of the imaging techniques used in the study. A total of 16 samples were obtained, of which 15 contained DMs forming the positive control group, while one area without DMs served as the negative control. The materials used were as follows:

1. Composite (Palfıque, Tokuyama Dental Corporation, Tokyo, Japan).
2. Amalgam (Ruby Dent, Istanbul, Türkiye).
3. Glass-ionomer cement (GIC) (Nova Glass GL, Nova Resins, Belgrade, Serbia).
4. Zinc polycarboxylate cement (Adhesor Carbofine, Spofa Dental, Germany).
5. Gutta-percha #40 (Dentplus, Choonchong, Korea).
6. Paper point #35 (Dentplus, Choonchong, Korea).
7. Cold-curing acrylic (Imicryl SC; Imicryl Dental Materials, Inc., Konya, Türkiye).
8. Wax (Polywax, Bilkim Kimya San. Ltd., İzmir, Türkiye).
9. Syringe with hypodermic needle 0.40 mm x 50 mm 27 G (Berika, Berika Medical Technology, Konya, Türkiye).
10. Stainless steel bracket #24 (0.018-inch slot, Master Series^®^,  American Orthodontics, Sheboygan, Wisconsin, USA).
11. Ceramic bracket #24 (0.018-inch slot, Radiance Plus^®^, American Orthodontics, Sheboygan, Wisconsin, USA).
12. Ni-Ti wires (NT3^®^ SE NiTi, 0.016 × 0.022-inch wire, American Orthodontics, Sheboygan, Wisconsin, USA).
13. Alginate (Tropicalgin Alginate, Zhermack SpA, Italy).
14. Acrylic tooth (Eray, Eraylar AŞ., Ankara, Türkiye).
15. Metal ceramic crowns (Shark MC porcelain, Zerodent, İstanbul, Türkiye).

Although the DMs were not standardized in shape, the dimensions of each material was recorded to provide descriptive information and is presented in Table 5. This range represents the variability encountered in clinical settings, which was intentionally preserved to test the diagnostic accuracy of CBCT and USG under conditions similar to those observed in practice.

### Radiographic and ultrasonographic examination

CBCT images of the samples were obtained using a Veraviewepocs 3D R100/F40 tomography device (J. Morita Mfg. Corp., Kyoto, Japan) at 90 kVp, 5 mA, and with a voxel size of 0.125 mm in an 8 × 10 cm field of view (FOV). The CBCT images were evaluated using i-Dixel 2.0 software (J. Morita Corporation, Osaka, Japan). The device was not equipped with a metal artifact reduction feature. The samples were stabilized using the standard head positioning accessories (chin support and headrest) of the device. Ultrasonographic examinations were performed using a MyLab™ Twice USG device (Esaote SpA, Genoa, Italy). A hockey-stick probe (IH 6–18, center frequency: 14 MHz) was employed. The imaging depth was set to 30 mm, the focal point to 10 mm, the gain to 50 dB, and the dynamic range to 12. The time-gain compensation (TGC) was kept constant at a medium level, and no additional standoff was applied. The grayscale level was fixed at the device’s default 256-level mode (midpoint: 128). Ultrasound examinations were conducted with the probe covered in ultrasound gel and stretch film (Fig. 1).

The images were obtained by an operator (Mİ), a dentomaxillofacial radiologist with 13 years of experience. Since the study was conducted in vitro, static images were preferred for USG.

### Assessment of radiographic images

The images were displayed on a 23-inch EIZO RadiForce MS230W Class Color LCD flat-panel screen (Eizo Nanao Corporation, Ishikawa, Japan). All images were evaluated in a darkroom, with observation conditions standardized by using the same computer monitor for all images.

All inter- and intra-observer evaluations were compared with the gold standard. The gold standard was created with intraoperative notes taken by the research assistant (EYK) during sample preparation. The observers (four and seven years of experience; ÇŞ, GG) were not involved in sample preparation or image acquisition, ensuring they were blinded to the “gold standard” (intraoperative records) and the obtained images. They were only informed that DM might be present in the soft tissue, but the type, number, or location of the material was not disclosed. The images were evaluated in a randomized order. After a washout period of at least four weeks, the datasets were re-read by two radiologists to assess intra- and inter-observer variability.

All samples were evaluated randomly for the presence or absence of DM and scored using the following 2-point scale: 0 = absence (the material could not be visualized in the image), 1 = presence (the material was clearly visualized in the image). This evaluation was performed individually for each DM rather than providing an overall “present/absent” judgment. In addition to the two-point detectability scoring system, further qualitative assessments were performed to characterize imaging features. Samples were classified as “opaque” if they exhibited a distinct bright opacity on the X-ray, “slightly opaque” if they had a faint radiographic presence, or “not visible” if they were not discernible on the image. Ultrasonographic echogenicity (isoechoic, hyperechoic, hypoechoic, anechoic) was assessed, and the presence of artifacts was noted (see Figs. 2 and 3).

### Statistical analysis

Statistical analysis was performed using SPSS version 22.0 (IBM Corp., Armonk, NY, USA). Intraobserver and interobserver agreements were evaluated using Cohen’s Kappa analysis, interpreted as follows: κ ≤ 0.20, poor; κ = 0.21–0.40, fair; κ = 0.41–0.60, moderate; κ = 0.61–0.80, good; and κ = 0.81–1.00, very good, with *p* < 0.05 considered statistically significant. Kappa values were calculated as unweighted since all measurements were based on nominal categories (present/absent), and 95% confidence intervals were determined using the Clopper–Pearson (exact) method.

Diagnostic accuracy parameters, including sensitivity, specificity, positive predictive value (PPV), negative predictive value (NPV), and overall diagnostic accuracy, were calculated for each imaging modality using 2 × 2 contingency tables that compared the imaging findings against the gold standard (presence or absence of DM). Sensitivity was defined as TP/(TP + FN), specificity as TN/(TN + FP), PPV as TP/(TP + FP), NPV as TN/(TN + FN), and overall diagnostic accuracy as (TP + TN)/(TP + TN + FP + FN), where TP, TN, FP, and FN represent true positives, true negatives, false positives, and false negatives, respectively. These calculations provided a comprehensive evaluation of the performance of CBCT and USG in detecting DMs embedded in soft tissues.

### Ethics approval statement

The sheep head used in this study was obtained from an animal slaughtered for human consumption and was provided as a donation from a local butcher. No experimental procedures were performed on living animals. According to the institutional policy of Zonguldak Bülent Ecevit University, research using animal tissues sourced post-mortem for human consumption does not require ethical approval.

## Conclusion

The findings of this study demonstrate that USG and CBCT offer distinct yet complementary advantages in imaging DMs within soft tissues. USG was highly accurate in detecting low-radiopacity, superficially located materials (e.g., paper points, wax, acrylic, and alginate) and differentiated material types through characteristic ultrasonographic artifacts. CBCT, on the other hand, effectively evaluated high-density materials (e.g., amalgam, metal-ceramic crowns, orthodontic brackets, and wires), but was insufficient for detecting low-density materials. These results suggest that selecting the appropriate imaging modality based on the material’s type and location can improve diagnostic accuracy, reduce unnecessary radiation exposure, enhance patient safety, and provide valuable information for clinical decision-making.

## Supplementary Information

Below is the link to the electronic supplementary material.

Supplementary Material 1

## Data Availability

Most of the data generated or analyzed are included in the article. The remaining datasets used and/or analyzed during the current study are available from the corresponding author upon request.
