# Supplementary figures and images for: In vitro comparison of the imaging properties of dental materials in soft tissue using ultrasonography and cone beam computed tomography
Source: Sci Rep. 2025 Dec 29;15:44928. doi: 10.1038/s41598-025-29235-4 (PMC12749840; doi:10.1038/s41598-025-29235-4)

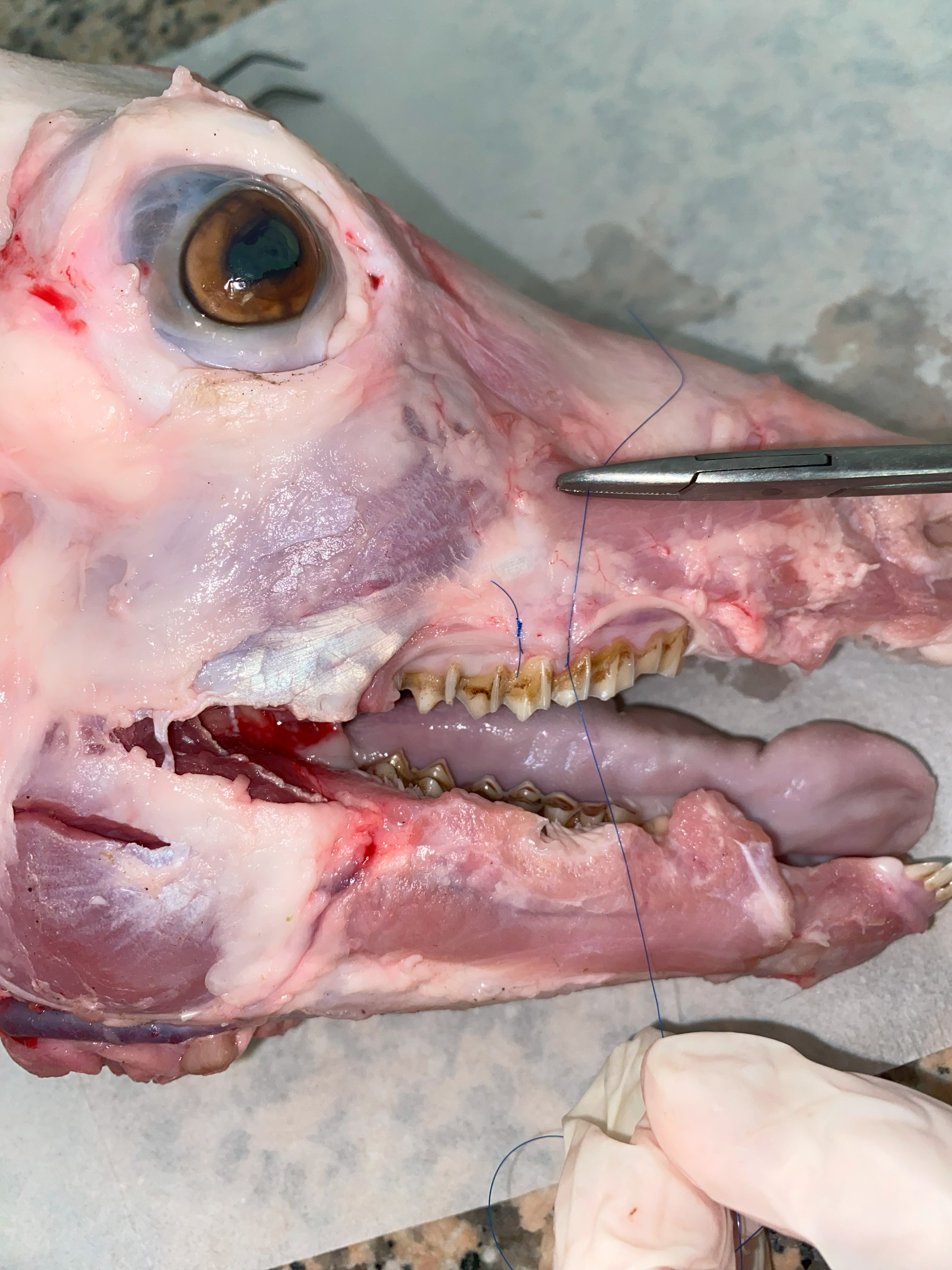

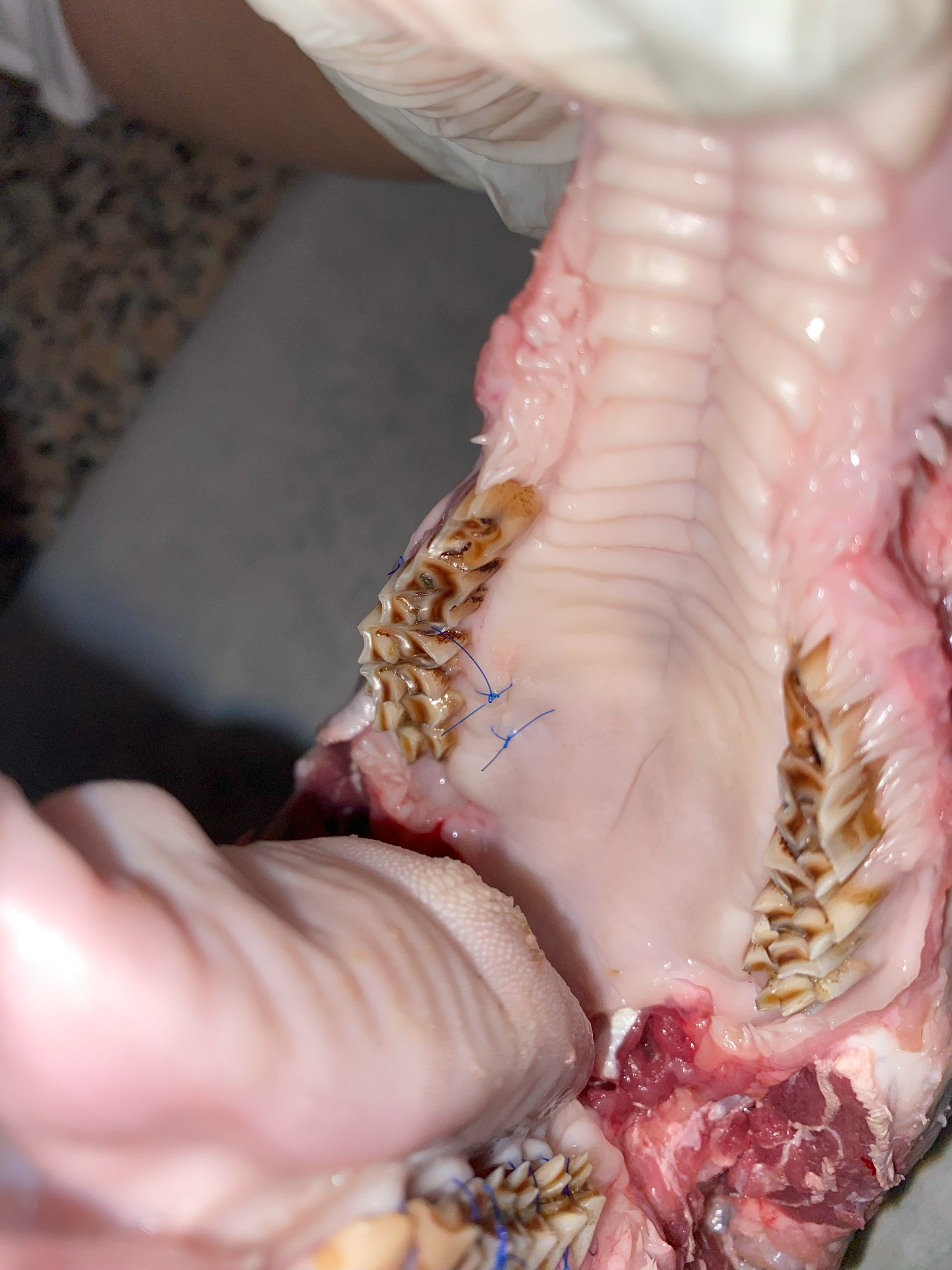

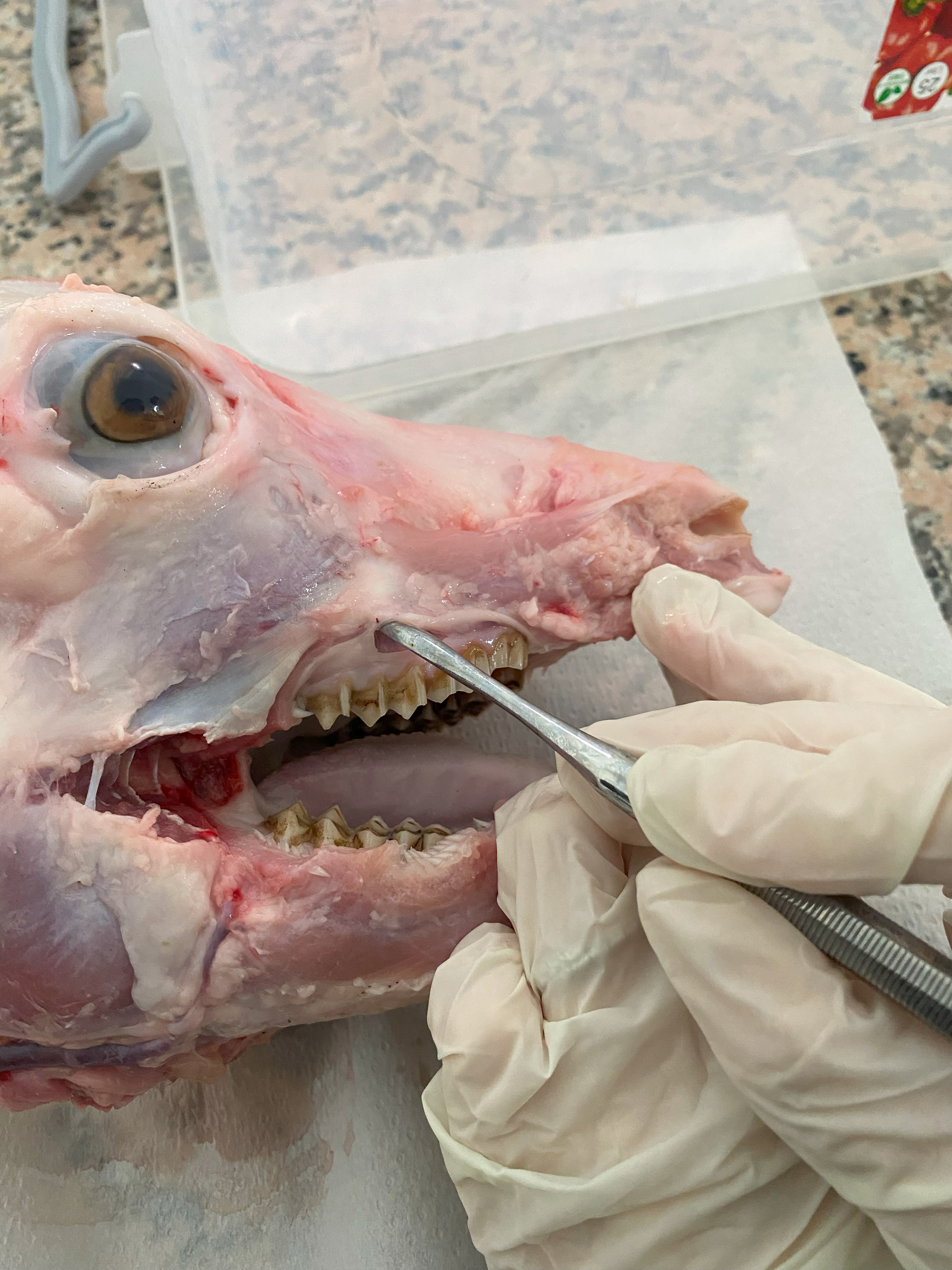

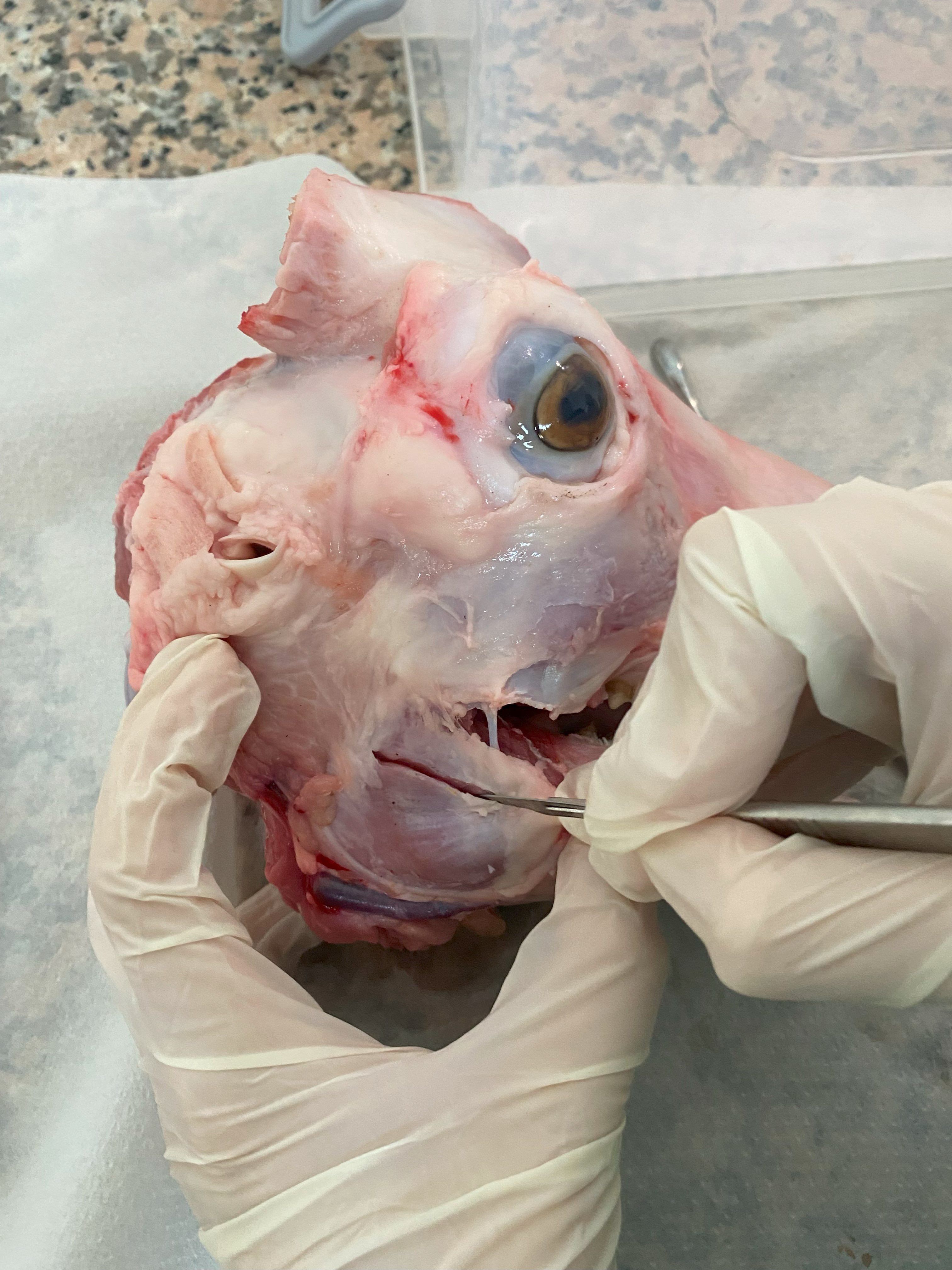

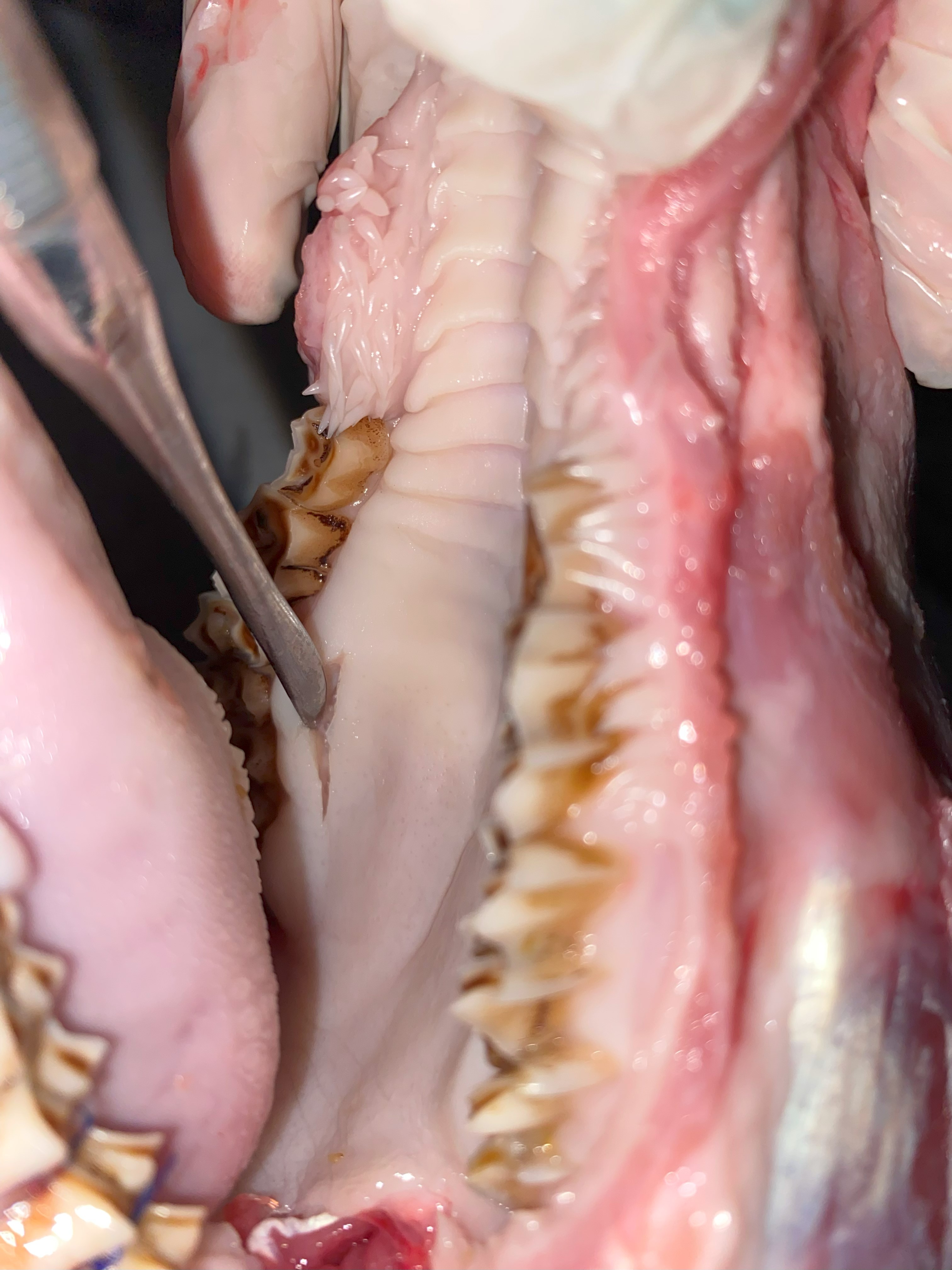

Supplement: Supplementary file 1 — Supplementary Material 1 [file 41598_2025_29235_MOESM1_ESM.docx]
